# Real-world treatment patterns and outcomes of chemo-immunotherapy with or without radiotherapy in extensive-stage small cell lung cancer: the durvalumab expanded access cohort

**DOI:** 10.3389/fonc.2026.1821628

**Published:** 2026-07-23

**Authors:** Urska Janzic, Walid Shalata, Mojca Unk, Ana Sophie Terglav, Natali Shirron, Sameh Daher, Mor Moskovitz, Ofer Merimsky, Patricia Garrido, Miguel Lopes, David Araujo, Talia Shantzer, Hadas Gantz-Sorotsky, Ofer Rotem, Damien Urban, Izabela Chmielewska, Ana Barroso, Alona Zer

**Affiliations:** 1 Department of Medical Oncology, University Clinic Golnik, Golnik, Slovenia; 2 Faculty of Medicine, University of Ljubljana, Ljubljana, Slovenia; 3 The Legacy Heritage Cancer Center and Dr. Larry Norton Institute, Soroka Medical Center, Beer Sheva, Israel; 4 Faculty of Health Sciences, Ben Gurion University of the Negev, Beer Sheva, Israel; 5 Division of Medical Oncology, Institute of Oncology Ljubljana, Ljubljana, Slovenia; 6 Division of Oncology, Rambam HealthCare Campus, Technion – Israel Technology Institute, Haifa, Israel; 7 Davidoff Cancer Center, Rabin Medical Center (MC), Petach Tikva, Israel; 8 Department of Bone and Soft Tissue Tumors, Tel Aviv Sorasky Medical Center, Tel Aviv, Israel; 9 Lung Cancer Unit, Champalimaud Foundation, Lisboa, Portugal; 10 Pulmonology Department Hospital Garcia de Orta, Almada, Portugal; 11 Pulmonology Department, Centro Hospitalar Universitário São João, Porto, Portugal; 12 Jusidman Cancer Center, Sheba Medical Center, Ramat Gan and the Faculty of Medical and Health Sciences, Tel Aviv University, Tel Aviv, Israel; 13 Department of Pneumonology, Oncology and Allergology, Medical University of Lublin, Lublin, Poland; 14 Multidisciplinary Thoracic Tumors Unit, Centro Hospitalar de Vila Nova de Gaia/Espinho, Vila Nova Gaia, Portugal

**Keywords:** chemo-IO, consolidation radiotherapy, ES-SCLC, palliative radiotherapy, salvage radiotherapy

## Abstract

**Purpose:**

To describe real-world treatment patterns and outcomes among patients with extensive-stage small cell lung cancer (ES-SCLC) treated with first-line chemotherapy plus durvalumab as in CASPIAN trial within Expanded Access Program, with a focus on the use and impact of consolidation (cRT) and salvage radiotherapy (sRT).

**Patients and methods:**

This retrospective, multicenter cohort study included patients with ES-SCLC treated with durvalumab plus platinum–etoposide between September 2019 and December 2022 across 11 academic centers in Israel, Poland, Portugal, and Slovenia. Patients characteristics, radiotherapy use, adverse events (AE), and outcomes were collected using a standardized form. Radiotherapy was categorized as cRT, sRT, or palliative RT (pRT). Progression-free survival (PFS) and overall survival (OS) were estimated using Kaplan–Meier methods and compared using log-rank testing. Multivariable Cox regression evaluated predictors of survival.

**Results:**

A total of 133 patients were included (median age 65 years; 74% ECOG PS 0–1). Radiotherapy was administered to 58.6% of patients (cRT 28.2%, sRT 24.3%, pRT 47.4%). Median (m) PFS and mOS for the entire cohort were 6.9 months (m) and 15.9 m, respectively. Patients receiving cRT achieved the longest mPFS (11.7 m) and a mOS of 19.5 m; sRT was associated with an mOS of 20.4 m. Continuation of durvalumab beyond progression combined with RT was associated with improved survival (p = 0.024). Treatment-related AEs occurred in 38.3% of patients, immune-related toxicities were infrequent, and pneumonitis was rare.

**Conclusion:**

In this multinational real-world cohort, outcomes with first-line chemo-immunotherapy were consistent with CASPIAN trial. The addition of cRT or sRT appeared feasible, safe, and associated with clinically meaningful survival improvements. Prospective trials are needed to define optimal integration of radiotherapy with chemo-immunotherapy in ES-SCLC.

## Introduction

Extensive-stage small cell lung cancer (ES-SCLC) is still a disease with limited treatment options and very poor survival. SCLC comprises an estimated 250.000 new cases globally each year and results in at least 200.000 deaths (1, 2). It is an aggressive disease characterized by rapid growth and metastatic spread (3). It accounts for about 10 – 15% of all lung cancers and tobacco remains the principal cause of SCLC, thus the disease could be largely prevented through effective tobacco control policies, since the vast majority of SCLC cases are missed even through effective lung cancer screening programs (4).

Until recently, the standard treatment for ES-SCLC relied solely on chemotherapy, based on the pivotal studies from the 1980’s, resulting in a median survival of under a year (5–7). Although patients with SCLC initially showed promising responses to platinum-based chemotherapy, these responses were brief because of the development of drug resistance (8, 9). The addition of immune checkpoint inhibitors (IO), including durvalumab and atezolizumab, to chemotherapy has emerged as the standard of care for ES-SCLC in recent years, based on two clinical trials that showed significant benefit (10, 11). Durvalumab added to platinum based chemotherapy resulted in 2.4 months overall survival (OS) gain (12.9 vs 10.5 months; HR 0.71 [95% CI 0.60-0.86]; p = 0.0003) and 36-month OS rate of 17.6% vs. 5.8% with chemotherapy alone. Moreover, there is a tail of the curve evident with about 10% of patients achieving long-term benefit that extends 3 years (12). Atezolizumab showed similar benefit with mOS of 12.3 vs. 10.3 months (HR 0.70; [95% CI 0.54 - 0.91]; p = 0.007) (11).

The landmark trials establishing chemo-immunotherapy for ES-SCLC, excluded the use of consolidation radiotherapy (cRT) or salvage radiotherapy (sRT) following the induction chemotherapy phase or when treating oligo-progression while continuing IO treatment. While prospective trials are evaluating the use of cRT with chemo-immunotherapy treatment for ES-SCLC (NCT06223711, NCT05544149), these are still ongoing and only limited real-world data are available from retrospective cohorts regarding the outcome and benefit of radiotherapy after chemo-immunotherapy in ES-SCLC (13–21).

We aimed to evaluate treatment patterns and outcomes of patients with ES-SCLC in the real-world setting treated with platinum-based chemotherapy and durvalumab within an Expanded Access program that was available in several European countries with an emphasis on the use of non-palliative radiotherapy either as cRT or sRT.

## Patients and methods

### Study design and population

This is a real-world retrospective, multicentric, observational study, utilizing data collection from medical records or applicable registries of patients with ES-SCLC, who underwent treatment with durvalumab plus platinum-based chemotherapy within the CASPIAN Expanded Access Program (EAP) from September 2019 until December 2022. Data on clinical and demographic characteristics as well as treatment patterns were collected from hospital medical records on a pre-specified electronic case report form (e-CRF) in eleven academic centers in four countries (Israel, Poland, Portugal, Slovenia). Radiotherapy intent was categorized as consolidation radiotherapy (cRT), salvage radiotherapy (sRT), palliative radiotherapy, or prophylactic cranial irradiation (PCI). Consolidation radiotherapy (cRT) was documented when radiotherapy was administered after completion of induction chemo-immunotherapy to residual thoracic disease in the absence of progression. Salvage radiotherapy (sRT) was defined as radiotherapy delivered during durvalumab maintenance for oligo-progressive disease in a single site, provided that durvalumab treatment was continued beyond progression. Prophylactic cranial irradiation (PCI) administration was also recorded. Dose and fractionation followed institutional standards and reflected the clinical intent of treatment. Palliative radiotherapy most commonly used short hypofractionated regimens (e.g., 8–20 Gy), whereas consolidation radiotherapy was typically delivered with intermediate doses, most frequently 30 Gy and occasionally 37.5–45 Gy. Salvage radiotherapy demonstrated the greatest heterogeneity, with doses ranging from 20 Gy to 60–66 Gy in selected cases, depending on disease site and prior treatment exposure.

Response to treatment was assessed and categorized as complete response (CR), partial response (PR), stable disease (SD) and progressive disease (PD), adapted from the Response Evaluation Criteria for Solid Tumors (RECIST) version 1.1. Radiologic response was evaluated according to the standard of care at each participating institution, with the first assessment typically performed after the second or third cycle of chemo-immunotherapy, which represents the routine and clinically appropriate timing for early response evaluation in ES-SCLC. Adverse events were assessed and graded based on the Common Terminology Criteria for Adverse Events (CTCAE) v 5.0. The study was approved by the local Institutional Review Boards in all participating centers, and the need for informed consent was waived.

### Statistic considerations

Missing data were addressed prior to statistical modeling; for categorical variables, if ≤5% of values were missing, imputation was performed using the mode. For > 5% missing data (notably in ECOG PS), imputation was performed using a Random Forest Classifier, trained on available numeric and categorical predictors. For numeric variables, missing values were imputed using the median. A Chi-square test was used to assess the association between the binary variable durvalumab beyond progression and treatment groups (ConsorSalv). A Kruskal–Wallis test was applied to compare AE counts between ConsorSalv groups due to the ordinal nature and non-normal distribution of the data. Progression-free survival (PFS) and OS were analyzed using the Kaplan–Meier method. PFS was measured from the date of systemic therapy initiation to the date of radiographic progression, death, or last follow-up. OS was measured from the diagnosis date to the death date or last follow-up. Patients without an event were censored at last follow-up. Censoring date was 19.06.2023. Median survival times and 95% confidence intervals (CI) were reported. Survival curves between treatment groups were compared using the log-rank test. A multivariable Cox proportional hazards model was constructed to estimate hazard ratios (HR) for PFS. Predictor variables included Sex (male/female), ECOG PS (0, 1, 2+), and Age <65. The model was stratified by ConsorSalv to control for treatment group effects. Hazard ratios with 95% CIs and p-values were reported.

## Results

This analysis included 133 patients with ES-SCLC who received first line treatment with durvalumab plus platinum-based chemotherapy between September 2019 and December 2020, in 11 centers across four countries (Israel, Poland, Portugal, Slovenia). There were 70% male patients, the median age was 64 years and 74% had an ECOG performance status (PS) of 0-1. Patient characteristics are presented in **Table 1**. Seven patients had known brain metastases at diagnosis, and all were irradiated before systemic treatment started. All patients received at least two cycles of chemotherapy with platinum (81% of patients received carboplatin) and etoposide, with a median of four cycles of chemotherapy doublet. The median time on durvalumab was 5.2 months (range 0 – 32.7 months). Best response to initial treatment was CR, PR, SD and PD in 5.6%, 58.6%, 11.3% and 11.3%, respectively and 13.2% of patients were not evaluable, which makes up to an overall response rate of 63.8% and disease control rate of 75.1%. Almost 82.0% of patients received chemotherapy treatment as initially planned, 6.0% ended due to disease progression or death and 3.0% due to treatment related adverse events.

**Table 1:** Patients baseline characteristics and treatment patterns.

Number of patients	133
Gender (%)	
Male	93 (69.9%)
Female	40 (30.1%)
Age, median (range)	64 (39 – 83)
ECOG PS (%)	
0-1	98 (73.7%)
≥ 2	27 (20.3%)
Unknown	8 (6.0%)
Stage at diagnosis (%)	
LS-SCLC	9 (6.8%)
ES-SCLC	124 (93.2%)
Smoking status (%)	
Never	4 (3.0%)
Former	17 (12.8%)
Current	112 (84.2%)
Pack/years smoked	
Median (range)	45 (0 – 150)
Chemotherapy regimen (%)	
Carboplatin – etoposide	108 (81.2%)
Cisplatin – etoposide	25 (18.8%)
Number of chemotherapy cycles, median (range)	4 (2 – 6)
Reason for chemotherapy discontinuation (%)	
Fulfilled 4–6 cycles	109 (82.0%)
Death or progressive disease	8 (6.0%)
Toxicity	4 (3.0%)
Unknown	12 (9.0%)
Time on durvalumab treatment, median (range)	5.3 months (0 – 37.2)
Best response to treatment	
CR	7 (5.6%)
PR	78 (58.6%)
SD	15 (11.3%)
PD	15 (11.3%)
Not evaluable	18 (13.2%)

ECOG PS, eastern cooperative oncology group performance status; LS-SCLC, limited stage small cell lung cancer; ES-SCLC, extensive stage small cell lung cancer.

### Treatment patterns

There were 78 patients (58.6%) who received radiotherapy during their first line treatment, either as consolidation radiotherapy (cRT), salvage radiotherapy (sRT) or palliative radiotherapy (pRT) in 28.2%, 24.3% and 47.4%, respectively, as shown in **Table 2**. Patients were paused on receiving IO during their RT treatment. The median duration of durvalumab treatment differed according to radiotherapy exposure: patients who did not receive radiotherapy had a median treatment duration of 4.5 months, compared with 7.6 months in those receiving consolidation radiotherapy (cRT), 5.4 months in those receiving salvage radiotherapy (sRT), and 4.6 months in those treated with palliative radiotherapy (pRT). Only two patients out the whole cohort received PCI.

**Table 2:** Details on radiotherapy received by patients with ES-SCLC treated with first line chemo-immunotherapy.

Radiotherapy received, no (%)	
Yes	78 (58.6%)
No	55 (41.4%)
Type of radiotherapy, no (%)	
Consolidation (cRT)	22 (28.2%)
Salvage (sRT)	19 (24.3%)
Palliative (pRT)	37 (47.4%)
Time of durvalumab treatment according to radiotherapy, median [months]	
No RT	4.5 (95% CI 5.23 – 8.93)
cRT	7.5 (95% CI 6.75 – 10.60)
sRT	5.4 (95% CI 3.96 – 7.90)
pRT	4.6 (95% CI 4.35 – 7.84)

RT, radiotherapy; cRT, consolidation radiotherapy; sRT, salvage radiotherapy; pRT, palliative radiotherapy.

A total of 42 patients continued durvalumab beyond progression and also underwent radiotherapy. In accordance with the predefined definitions, radiotherapy in this setting was delivered predominantly as salvage radiotherapy (sRT) for oligo-progressive disease during durvalumab maintenance, while a smaller proportion received palliative radiotherapy depending on clinical presentation. Patients who received radiotherapy while continuing durvalumab beyond progression demonstrated a statistically significant improvement in survival compared with those who did not receive radiotherapy (p = 0.024; **Supplementary Figure 1**).

At data cut-off, 14 (10%) of patients were still receiving durvalumab maintenance therapy. Out of the whole cohort, 54 (41%) of patients proceeded to 2^nd^ line systemic therapy, mostly chemotherapy (94%), two patients received a combination of PD-1 and CTLA-4 inhibitors and one patient was enrolled in a clinical trial. Third-line treatment was administered in 20 (15%) of patients.

### Treatment outcomes

**Figure 1:**
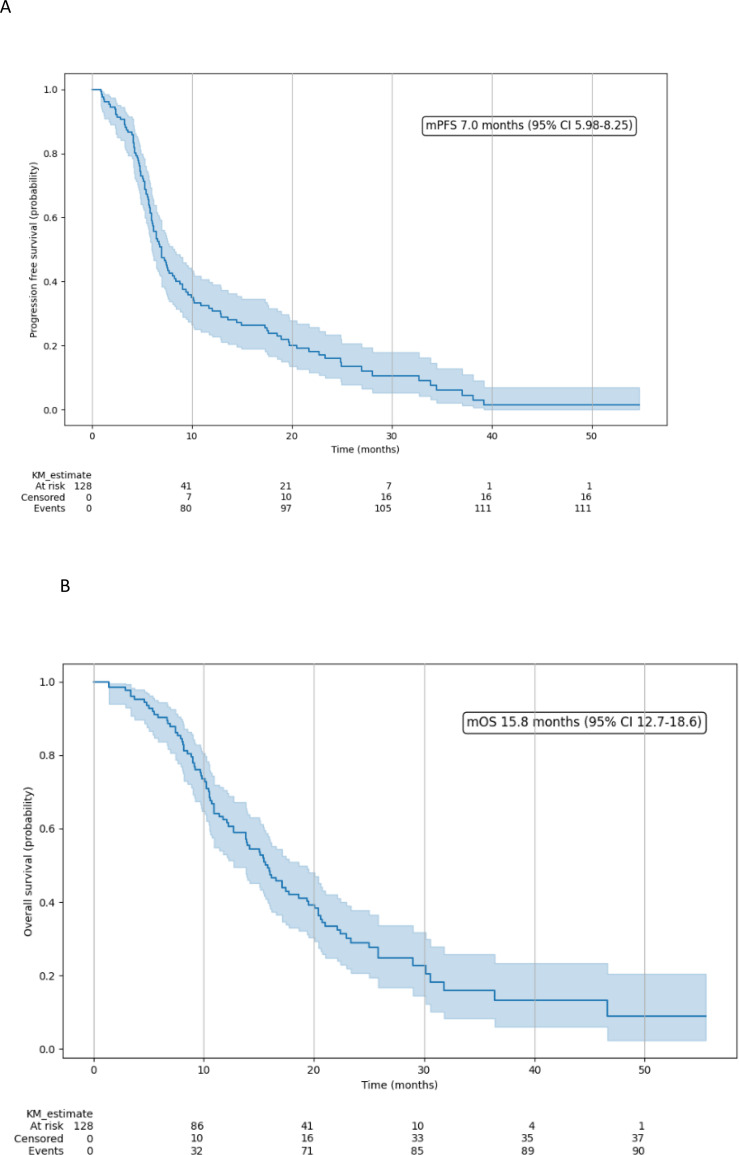
**(A)** Kaplan-Meier curves of progression free survival (left) and **(B)** overall survival (right) of the entire cohort of treated patients. Broader lines are showing 95% confidence intervals. mPFS, median progression free survival; mOS, median overall survival; CI, confidence intervals.

**Figure 2:**
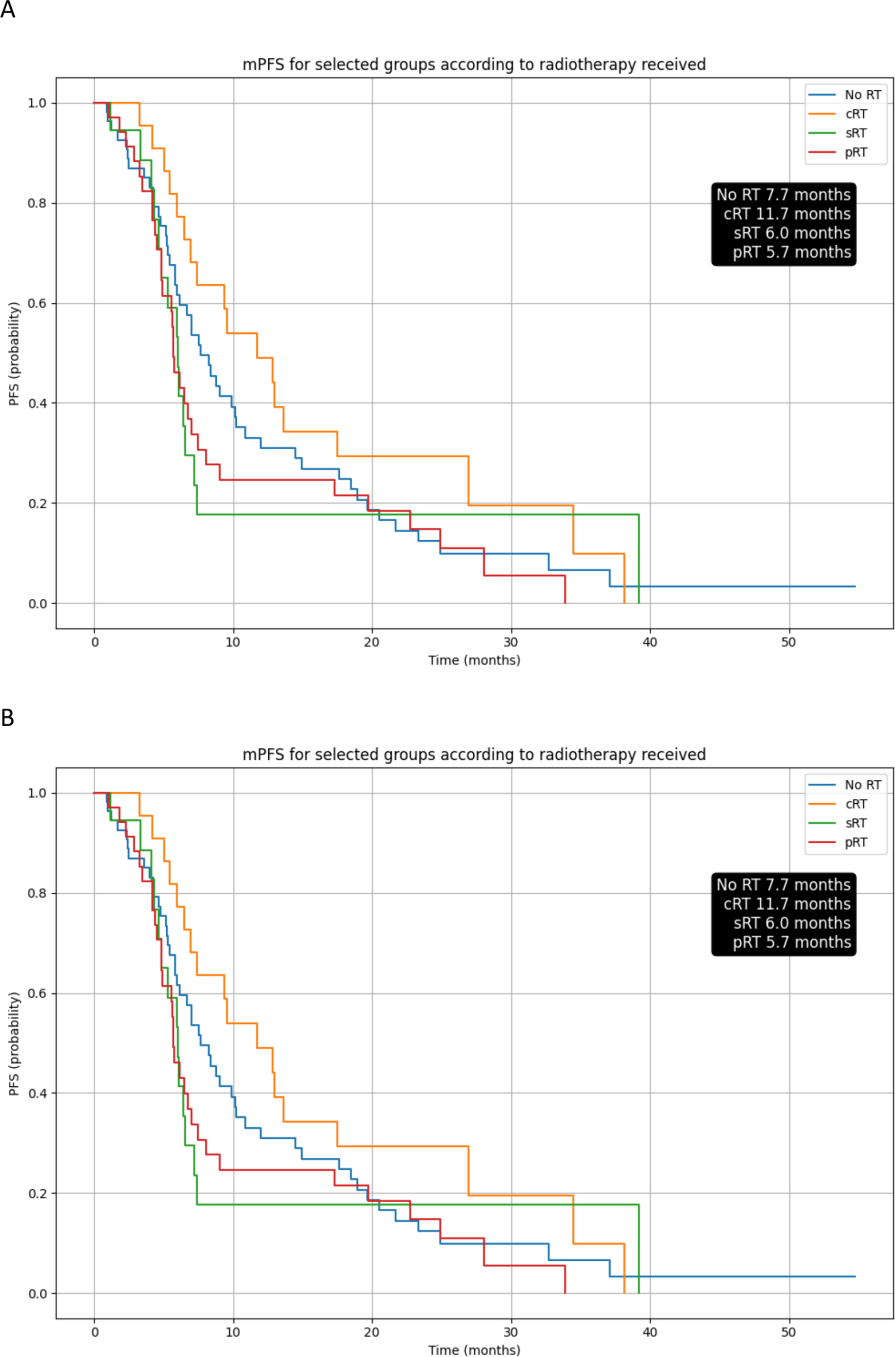
**(A)** Progression free survival (above) and **(B)** overall survival (bellow) of patients with ES-SCLC treated with first line chemo-immunotherapy and receiving either no or one of the radiotherapy options (cRT, consolidation radiotherapy; sRT, salvage radiotherapy; pRT, palliative radiotherapy).

With a median follow-up of 19.8 months, the median progression-free survival (mPFS) was 6.9 months for the whole cohort of patients (95% CI 5.98 – 8.25) and mOS was 15.9 months (95% CI 13.8 – 19.5). Patients receiving cRT had the longest mPFS of 11.7 months and an OS of 19.5 months (95% CI 13.9 – 36.4). Patients receiving sRT had a mPFS of 5.72 months and a mOS of 20.4 months (95% CI 10.5 – ND). As expected, patients in good ECOG PS 0–1 had meaningly longer mOS than those with ECOG PS ≥ 2 with 13.3 months (95% CI 1.37 – 47.10) versus 10.3 months (95% CI 1.40 – 25.27; p = 0.028). Median PFS and OS survival curves are shown in **Figures 1** and **2**.

In the multivariable Cox regression analysis for overall survival, no covariates demonstrated statistically significant associations with outcome. Age under 65 years showed the strongest effect, with an HR of 0.63 (95% CI 0.41–0.97; p = 0.03), while consolidative radiotherapy demonstrated a non-significant trend toward improved overall survival compared with no thoracic radiotherapy (HR 0.58; 95% CI 0.32–1.07; p = 0.08).

### Safety

Treatment – related adverse events of any grade were reported in 51 patients (38.3%). There were no significant differences in terms of adverse event recording between patients being treated with no RT, cRT, sRT or pRT, (p = 0.599) (**Supplementary Table 1**). Severe adverse events were scarce and rarely led to treatment interruption or cessation. Discontinuation of durvalumab due to immune-related adverse events was reported in six patients, experiencing pneumonitis, allergic reaction, uveitis, ataxia, colitis and one patient both pneumonitis and colitis. Off note, the first patient with pneumonitis did not receive any thoracic irradiation and the second received consolidation radiotherapy before maintenance durvalumab. Another case of milder pneumonitis (G1) was reported in a patient that also received palliative radiotherapy to the lungs and bone, but that patient continued treatment with durvalumab without further complications. There was one treatment-related death reported with a patient experiencing exacerbation of neurologic symptoms, probably due to worsening of paraneoplastic neurologic disorder during durvalumab treatment.

## Discussion

Patients in our real-world retrospective, multicentric, observational study exhibited comparable outcomes in terms of PFS and OS as in the pivotal CASPIAN trial, where mPFS was 5.1 months and mOS was 13.0 months, since the whole group of our real-world patients reached a mPFS of 7 months and mOS of 15.9 months (10, 12). Notably nearly 60% of patients in our study received radiotherapy at one point during treatment. However, even patients who did not receive radiotherapy achieved mPFS and mOS outcomes (7.7 and 14.1 months, respectively) comparable to those in the CASPIAN trial. While the integration of IO in ES-SCLC treatment has shown meaningful benefits, clinicians recognize that overall outcomes remain poor and are therefore exploring ways to improve them through the addition of radiotherapy (16).

Since ES-SCLC is a particularly chemo- and radiotherapy sensitive disease, it is reasonable to try to either combine these treatment modalities or use them sequentially any time during the treatment course (13). Several RCTs evaluated the role of consolidative thoracic RT in patients with ES-SCLC after platinum-based chemotherapy with conflicting OS results despite clear benefit in local control (22–25). A meta-analysis of 3 RCTs with 690 patients demonstrated a reduction in the risk of progression with cRT by 28% (HR 0.72; 95% CI 0.61–0.83; p < 0.0001), and significantly reduced chance of thoracic relapse (RR 0.52). Another systematic review of 12 trials (1,995 patients) confirmed significant benefits in PFS (HR 0.64) and local recurrence-free survival (HR 0.38), all with p < 0.00001. Both analyses failed to prove a significant benefit in terms of OS between the two groups with HR 0.61 (95% CI: 0.36–1.01, P = 0.06) and HR 0.88 (95% CI 0.66 - 1.18, p=0.36) (23, 26).

Most clinical trials investigating cRT were conducted in the pre-IO era, making it challenging to directly apply their findings to the current treatment landscape due to concerns of increase in adverse events, especially pneumonitis (22, 24, 25, 27–30). Even the European Society for Medical Oncology guidelines offer only vague recommendations, suggesting that cRT could be considered after induction chemo-IO treatment following a shared decision process with the patient (31). Still, emerging evidence, primarily from retrospective studies, and from a single phase I clinical trial, suggest that cRT or sRT) could potentially benefit patients with ES-SCLC without exposing them to further risk of serious adverse events (13–15, 17–21, 32, 33). The underlying rationale for combining chemo-IO is the possible induction of systemic anti-tumor immune responses. Radiotherapy can promote immunogenic cell death and may act synergistically with IO, potentially enhancing local control and systemic immunity. While careful monitoring for pneumonitis is necessary given the risk from both IO and cRT, current data indicate that cRTor sRT are generally well tolerated. The benefit of thoracic RT after chemo-IO in ES-SCLC patients in under prospective evaluation in the NRG LU007 clinical trials (NCT04402788).

Several retrospective studies reported the efficacy of cRT after chemo-IO in patients with ES-SCLC, three of them enrolling 211, 45 and 46 patients receiving cRT after chemo-IO and showed significant benefit both in PFS (HR 0.64; p=0.025) and OS (HR 0.52; p=0.015) in the first; PFS HR 0.59 (p=0.009) and OS HR 0.53 (p=0.016) in the second and PFS HR 0.44 (p<0.001) and OS HR 0.58 (p < 0.001) in the third trial, respectively (18, 20). The largest data-set so far coming from the United Kingdom and Switzerland, also confirms the benefit of PFS and OS for patients that did receive cRT with PFS HR 0.61 (95% CI: 0.48–0.77, p < 0.001) and OS HR 0.53 (95% CI: 0.4–0.71, p < 0.001) (21).

Our study contributes to accumulating data supporting the use of cRT and sRT in this scenario of ES-SCLC patients responding the Chemo-IO. While our data did not show an OS benefit with cRT and sRT we have observed a trend of 5-month gain in OS with cRT (19.5 months) and sRT (20.4 months) versus no RT (14.2 months) which is clinically meaningful for this group of patients.

Treatment with chemo-IO and cRT proved to be safe in our retrospective study, with about 38% of patients experiencing treatment related adverse events and only 6 patients discontinuing durvalumab due to immune-related adverse events and only two out of those experiencing pneumonitis, none being fatal. Patients receiving any form of radiotherapy and those that have not, had a similar rate of adverse events reported (p=0.599). In the UK-Swiss group, patients who received consolidation thoracic RT, had similar rate of irAEs (38%) with three cases of grade 3 toxicity but also two cases of fatal pneumonitis that were not reported in our cohort (21). Others reported up to 37% adverse events, mostly attributable to chemotherapy, the rate of all-grade pneumonitis was up to 10% with rare cases of severe or even fatal events (14, 17, 18, 20, 32).

We acknowledge some limitations of our study, such as its retrospective nature, which makes the analysis subject to variability in documentation quality and completeness across participating centers. We also recognize the possibility of selection bias, as patient inclusion was not randomized and may reflect institutional practice patterns. Additionally, the absence of a centralized review process for imaging, treatment planning, and outcome assessment introduces potential inter-observer variability. Finally, we note that methodological heterogeneity—particularly regarding radiotherapy techniques, dose specifications, and patient selection criteria—may influence the consistency of treatment parameters. Nevertheless, our findings reflect real-world treatment patterns and provide meaningful evidence of the potential benefit for patients treated with this approach.

In conclusion, continuing radiotherapy treatment after induction chemo-IO has proven to be both feasible and yields excellent results in several retrospective studies, including ours, which was multinational and had very few other biases, since patients had to be eligible to receive IO within the EAP. Furthermore, very few patients experienced severe adverse events, including pneumonitis, which was mostly not a treatment-limiting toxicity. Optimal timing and radiation dose and fractionation after chemo-IO are still being defined as maintenance IO treatment in case of consolidation radiotherapy. All these questions need to be studied further in prospective randomized trials to assure the optimal treatment sequence for patients with ES-SCLC where progress resulting in OS is extremely anticipated.

## Data Availability

The raw data supporting the conclusions of this article will be made available by the authors, without undue reservation.

## Supplementary material

Supplementary Table 1 Proportion of patients with ≥1 adverse event by treatment group:

Supplementary Figure 1 Treatment with durvalumab beyond progression according to radiotherapy received. No RT, no radiotherapy; cRT, consolidation radiotherapy; sRT, salvage radiotherapy; pRT, palliative radiotherapy.
