# Supplementary material for: Real-world treatment patterns and outcomes of chemo-immunotherapy with or without radiotherapy in extensive-stage small cell lung cancer: the durvalumab expanded access cohort
Source: Front Oncol. 2026 Jul 23;16:1821628. doi: 10.3389/fonc.2026.1821628 (PMC13441931; doi:10.3389/fonc.2026.1821628)
Supplement: Supplementary file 1 [file Table1.pdf]

Supplementary Table 1. Proportion of patients with ≥1 adverse event by treatment group:

**Treatment Group**	**0 AEs**	**≥1 AE**
No RT	33	22
Consolidation	15	7
Palliative	21	16
Salvage	13	6
